# Does the peer-led Honest, Open, Proud program reduce stigma’s impact for everyone? An individual participant data meta-regression analysis

**DOI:** 10.1007/s00127-023-02491-3

**Published:** 2023-05-09

**Authors:** Thomas Klein, Markus Kösters, Patrick W. Corrigan, Winnie W. S. Mak, Lindsay Sheehan, Colleen S. Conley, Nathalie Oexle, Nicolas Rüsch

**Affiliations:** 1 Section Public Mental Health, Department of Psychiatry II, Ulm University and BKH Günzburg, Parkstraße 11, 89073 Ulm, Germany; 2 Center for Evidence-Based Healthcare, Medical Faculty Carl Gustav Carus, Technische Universität, Dresden, Germany; 3 Department of Psychology, Illinois Institute of Technology, Chicago, IL USA; 4 Department of Psychology, The Chinese University of Hong Kong Shatin , NT, Hong Kong SAR; 5 Department of Psychology, Loyola University Chicago, Chicago, IL USA

**Keywords:** Self-stigma, Internalized stigma, Honest, Open, Proud, Coming Out Proud, Peer-led interventions, Meta-analysis

## Abstract

**Purpose:**

Many people with mental illness experience self-stigma and stigma-related stress and struggle with decisions whether to disclose their condition to others. The peer-led Honest, Open, Proud (HOP) group program supports them in their disclosure decisions. In randomized controlled trials, HOP has shown positive effects on self-stigma and stigma stress on average. This study examined individual predictors of HOP outcomes and tested the hypothesis that stigma stress reduction at the end of HOP mediates positive HOP effects at follow-up.

**Methods:**

Six RCTs were included with data at baseline, post (after the HOP program) and at 3- or 4-week follow-up. Baseline variables were entered in meta-regression models to predict change in self-stigma, stigma stress, depressive symptoms and quality of life among HOP participants. Mediation models examined change in stigma stress (post) as a mediator of HOP effects on self-stigma, depressive symptoms, and quality of life at follow-up.

**Results:**

More shame at baseline, and for some outcomes reduced empowerment, predicted reduced HOP effects on stigma stress, self-stigma, depressive symptoms, and quality of life. Younger age was related to greater improvements in stigma stress after the HOP program. Stigma stress reductions at the end of HOP mediated positive effects on self-stigma, depressive symptoms and quality of life at follow-up.

**Conclusion:**

Participants who are initially less burdened by shame may benefit more from HOP. Stigma stress reduction could be a key mechanism of change that mediates effects on more distal outcomes. Implications for the further development of HOP are discussed.

**Supplementary Information:**

The online version contains supplementary material available at 10.1007/s00127-023-02491-3.

## Introduction

Many people with mental illness internalize public prejudice and experience self-stigma as a consequence [1]. Self-stigma occurs if people agree with negative stereotypes and turn them against themselves (“I must be stupid, because I have a mental illness”). Self-stigma has serious negative effects in terms of poor clinical outcomes, suicidality, social isolation, lack of help-seeking, demoralization [2] and leads to a ‘why try’ effect if people give up to pursue their life goals [3]. Based on stress-coping models [4], a further consequence of public stigma is that many people with mental illness perceive stigma as a stressor if they feel that stigma-related harm exceeds their personal coping resources [5]. This so-called stigma stress predicts self-stigma in longitudinal studies [2, 6]; it is also related to poor clinical and social outcomes such as low quality of life, impaired recovery, suicidality and the transition from subclinical syndromes to psychosis [6–9]. Self-stigma and stigma stress are associated with secrecy, or the decision not to disclose one’s condition to others [10]. As a mental illness is not easily recognized by others, many find it hard to decide whether to disclose to people in their social environments—arguably a key decision in coping with public and self-stigma [11].

The Honest, Open, Proud (HOP) program, formerly known as Coming Out Proud (COP), is a peer-led group program that supports people with mental illness with their disclosure decisions. It is not HOP’s goal to make people disclose, but to let them weigh pros and cons of disclosure, depending on their social environment and their personal goals. HOP teaches ways to find addressees for a potential disclosure and ways to tell one’s story, should a person decide to do so. HOP is a compact program, usually delivered in three 2 h sessions, in more recent versions followed by a booster session about a month after the third session. The main topic of session 1 is to weigh pros and cons of disclosure in different settings. Session 2 covers different levels of disclosure, from social withdrawal and secrecy to indeterminate disclosure and broadcasting of one’s story; also in Session 2 are ways to find out who might be a good person to disclose to. In session 3 people learn to tell their story, in case they decide to do so. The fourth and booster session reflects participants’ experiences with disclosure as well as non-disclosure and revisits (non-)disclosure decisions and goals and ways to tell one’s story. For people with a history of suicide attempts or suicidality, there is a specific HOP version developed by Lindsay Sheehan and colleagues. It maintains the three core lessons of HOP, but includes disclosure stories and examples that depict suicide ideation and suicide attempts. The instructor manual includes guidelines for screening, assessing and managing disclosures of suicidal thoughts or behaviors within the group. This HOP version covers disclosure on social media, in the workplace and in educational settings, in addition to brief sections on empowered non-disclosure and coerced disclosure of suicidality. More information on other HOP versions is provided in a recent review (Table 1 in [12]).

A recent meta-analysis based on five randomized-controlled trials (RCTs) of HOP looked at average effects of HOP among people with mental illness or with a history of suicidality: it found significant moderate-sized positive effects on stigma stress after the three sessions as well as significant weak-sized effects on self-stigma at 3 week follow-up ([12], for an overview of peer-led interventions to reduce self-stigma in general see [13, 14]). It is, however, unclear whether individual characteristics, such as socio-demographic or stigma-related variables such as shame about one’s mental illess or lack of empowerment at baseline, predict HOP’s efficacy. A better understanding of outcome predictors would allow to offer HOP to those who are most likely to benefit—and to modify the program for others. It is further unknown whether reduced stigma stress, as a proximal outcome of HOP, predicts more distal outcomes such as improved quality of life (as it was the case in a trial of HOP for adolescents [15]).

This study therefore had two aims: First, to identify factors that influence HOP effects, using a meta-regressional approach based on raw individual participant trial data. Based on previous RCTs, we expected younger age [15] and female gender [16] to predict better outcomes. We used novel analytic approaches to explore outcome predictors on the study and individual level. The analysis of individual participant data (IPD, i.e. data for each participant from each trial) offers a sophisticated method to maximize precision of value estimation and the power to identify predictors of intervention outcomes [17]. Second and based on the above-mentioned previous trial [15], we expected that reduced stigma stress after the end of the HOP program would mediate HOP effects at follow-up on quality of life, self-stigma, and depressive symptoms.

## Methods

This meta-analysis was registered in the international Prospective Register of Systematic Reviews (PROSPERO: Nr. CRD42021271157). It was conducted following the PRISMA guidelines for individual participant data (Stewart et al., 2015) as far as applicable (see Supplementary Table S1). The meta-analysis was approved by the ethics committee of Ulm University (Nr. 345/18).

### Search strategy

We searched for HOP trials in English and German with the search terms “Honest, Open, Proud”, “Coming Out Proud” (HOP’s previous name; hopprogram.org), and “In Würde zu sich stehen” (the German name of HOP; uni-ulm.de/med/iws/) in the databases PubMed, MEDLINE and PsycINFO without time limits. Only randomized-controlled trials were included. Since HOP is a peer-led program, trials were included only if HOP groups were led or co-led by people with lived experience of mental illness (or, regarding the HOP version for suicide attempt survivors, by people with experience of suicidality). Members of the HOP International Steering Committee (Michelle Andra, Patrick W. Corrigan, Jon Larson, Melissa Pyle, Sang Qin, Nicolas Rüsch, Katrina Scior, Chris White) were also asked about published, or completed but yet unpublished, HOP trials they might be aware of.

### Data extraction

All data examined in this meta-analysis was kindly provided by the original study authors as full raw data sets. After all primary datasets of HOP trials had been assembled, completeness of the data as well as distributions of socio-demographic variables and scale scores were checked. In case of apparent inconsistencies between primary data and published results, authors of the primary studies were contacted to clarify misunderstandings. Data on the individual participant level were socio-demographic characteristics, stigma (e.g. stigma stress, self-stigma) and well-being (e.g. quality of life). Extractable study-level data included information on the trial itself (e.g. target group). The following study characteristics were extracted from primary datasets: trial location, stigmatized condition (mental illness or suicide attempt history), and type of scales used to assess moderators and outcome variables (including number of items and scaling). On the participant level, treatment condition (HOP vs control), age, gender, ethnicity, marital status, education level (less than high school, high school, college/university degree), and type of psychiatric diagnosis were extracted (if available; diagnoses were not specified in two trials [16, 18], and in the remaining four trials schizophrenia spectrum diagnoses were reported by 27% [19], 21% [20], 38% (Mak et al. unpublished Hong Kong trial) or, among adolescents, 4% [15], respectively). The following stigma- and health-related variables were extracted in the form of scale scores for all three assessment times, i.e. at baseline before the start of the HOP program (t0), after the end of HOP (post/t1, i.e. 3 weeks after baseline), and at follow-up (t2) three weeks [15, 18–20] or four weeks ([16] and the Hong Kong trial) after the end of HOP, i.e. 6 or 7 weeks after baseline: self-stigma, stigma stress, depressive symptoms, quality of life, empowerment, secrecy, and shame about having a mental illness (or a suicide attempt history).

### Outcome variables

The primary outcome measure for the purpose of this meta-analysis was the pre-post HOP effect size (as standardized mean difference/SMD) for self-stigma from baseline (t0) to the end of the HOP program (post/t1) as assessed by the apply subscale of the Self-Stigma of Mental Illness Scale-Short Form [21]. Secondary outcomes were effect sizes for depressive symptoms (Center for Epidemiologic Studies-Depression Scale [22]), quality of life (KIDSCREEN-10 [23] or Manchester Short Assessment of Quality of Life [24] or a 1-item question in [20]), and stigma stress which was measured by the Stigma Stress Scale and defined as the difference between the appraisal of mental illness stigma as harmful minus the appraisal of perceived resources to cope with stigma-related harm [5, 25]; higher difference scores indicate higher stigma stress. Detailed information on HOP outcome measures can be found elsewhere ([12], p. 1517, Table 2). SMDs from baseline to post (t0/t1) and from baseline to follow-up (t0/t2) were calculated for all outcomes.

### Risk of bias assessment

Two raters (TK, NR) evaluated the risk of bias in the included RCTs following the criteria of the Cochrane Collaboration risk of bias assessment (RoB tool 2.0 [26]). The RoB 2.0 tool assesses bias arising from randomization procedures, non-adherence to interventions as intended, and others. Although we had access to the IPD of all included trials, we evaluated bias related to missing outcome data and selective reporting of trial results, but not publication bias. For the other bias domains, the RoB was assessed based on the information provided in published trial reports and study articles. Possible sources of bias were judged to be of high, low, or unclear risk or raising some concerns. In case of disagreements, both raters discussed their evaluations to reach consensus and, if necessary, a third rater (MK) was involved.

### Statistical analyses

A one-stage meta-analytic approach was performed to combine all IPD from included studies in a single analysis using the metafor package [27] developed for use in the statistical environment R. Data were analyzed as if they belonged to a single ‘mega-trial’ in which participants were nested within studies. We performed a mixed-effect linear regression with random-effect intercepts model. The outcome was calculated as standardized mean difference (SMD) between the baseline and endpoint score for each outcome variable (t1 score – t0 score), whereby scores below zero indicate a decrease in the respective measure from baseline. In case different measures were used across trials for the same outcome domain, priority was given to the most commonly used measure. Scores were standardized by the scales’ maximum scores to be comparable with other measures for the same construct. The extent to which a moderator predicted the outcome score in the respective regression model was indicated by the regression coefficient b. The adjusted R^2^ indicates the total percentage of outcome variance explained by all included predictors, adjusted for the number of predictors in the model, and reflects the model’s overall goodness of fit. The last observation carried forward (LOCF) approach was used to impute missing values at t2 from t1 data for participants assessed post-intervention. Sensitivity analyses were conducted, from which imputed data for follow-up assessments had been excluded.

Based on our above-mentioned hypotheses, we tested younger age and female gender as predictors of better HOP outcomes in single-predictor meta-regression analyses. The other above-mentioned baseline variables were tested as predictors in exploratory regressions. In a second step, an analysis was conducted to identify predictors of HOP outcomes using the R-package GLMulti [28] to select the best model according to the Akaike Information Criterion [29] corrected for small sample sizes (AICc), which seeks to explain most variance of the dependent variable by the least number of predictors. Because model selection can fluctuate due to sample sizes and high numbers of comparisons, we took all tested models into consideration, not only the best one according to AICc [30], by estimating the relative importance of predictors across all tested models. To indicate the importance of a predictor for a specific outcome, the relative evidence weights of all models was summed in which the predictor appears [31]. Predictors were regarded as important for the specific outcome if they passed the established threshold of being included in 80% of tested models [28].

Finally, mediation analyses were conducted for regression models, in which stigma stress change at t1 (post) as a proximal outcome was tested as a mediator of HOP effects on three more distal outcomes (self-stigma, depressive symptoms, quality of life) at follow-up (t2), by using advanced structural equation modelling for meta-analyses ([32], see Fig. 4 for the examined path models).

## Results

### Characteristics of included studies

After the removal of duplicates, our literature search yielded 16 papers on HOP (or COP, its previous acronym), five of which were RCTs examining HOP as a peer-led intervention. Six HOP trials were included in this meta-analysis; five had been published [15, 16, 18–20] and one in Hong Kong, led by Winnie W. S. Mak, was completed, but yet unpublished. The trials had been conducted between 2012 and 2020 in Switzerland [19], the United States [16, 18, 20], Germany [15], or Hong Kong. All trials had the same assessment times: baseline (t0), post-HOP (t1) and a 3- or 4-week follow-up (t2). In total, 311 participants were randomized to the HOP program, and 328 to a treatment-as-usual control condition. Five HOP trials, with altogether 95% of HOP participants in this meta-analysis, included people with mental illness and one HOP trial was for suicide attempt survivors (5%). Except for one study among adolescents [15], 84% of HOP participants were adults between 18 and 72 years. HOP participants were on average 35.5 years old (SD = 16.3) and nearly two thirds (62%) were female (for further information see Supplementary Table S2). Similar to our recent aggregate meta-analysis [12], there were four outcome domains for which data was available: stigma stress, self-stigma and depressive symptoms from all six RCTs, and quality of life data from three trials.

### Results of meta-regression analyses between baseline (t0) and post-intervention (t1)

**Fig. 1:**
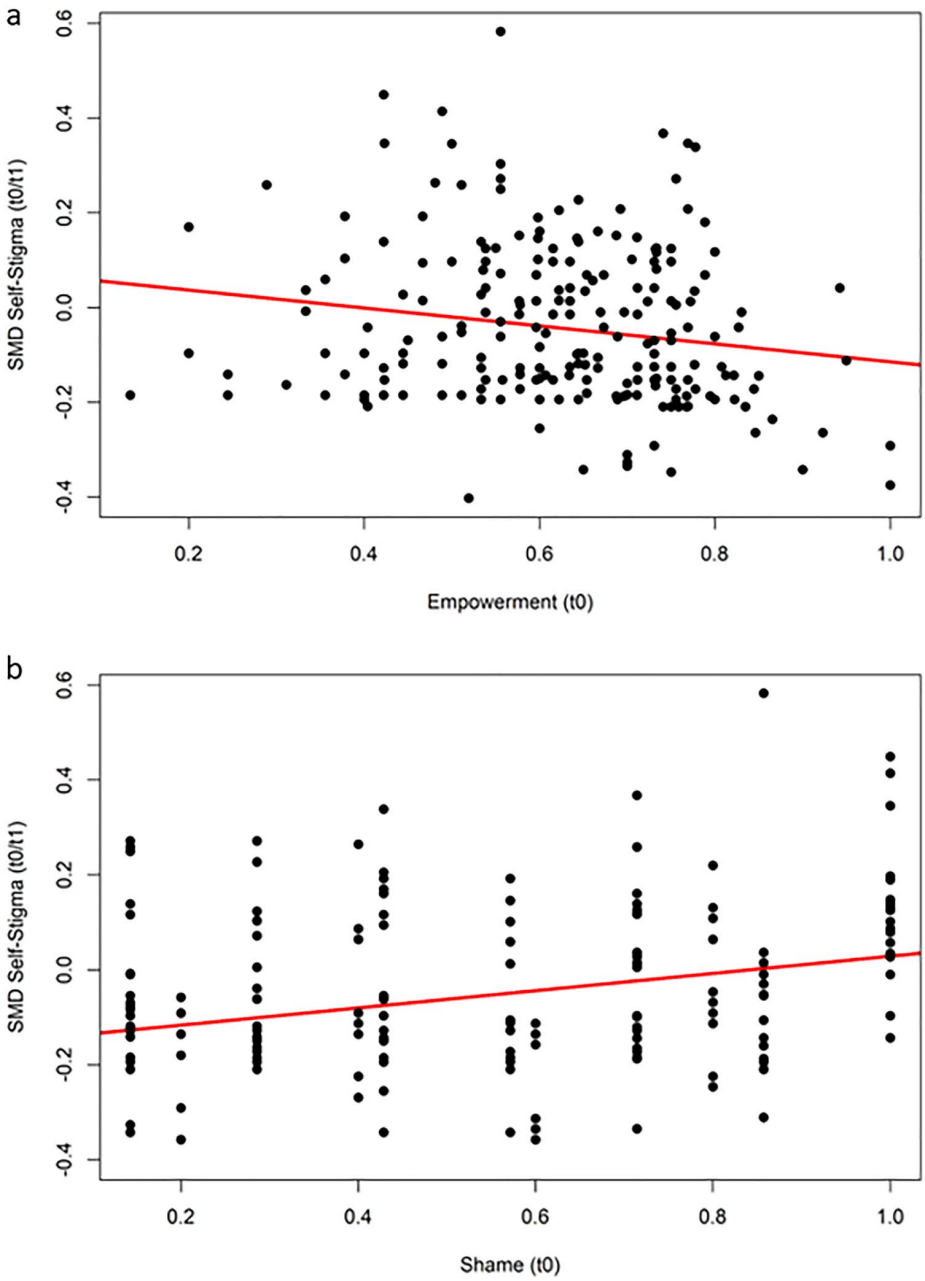
Two regressions on standardized mean difference (SMD) of self-stigma change (scores < 0 equalling self-stigma decrease) from baseline to end of the HOP program (pre-post, t0/t1), predicted by** a** empowerment or** b** shame, both at baseline. SMD scores on the y-axis below zero indicate a reduction of self-stigma at t1

**Fig. 2:**
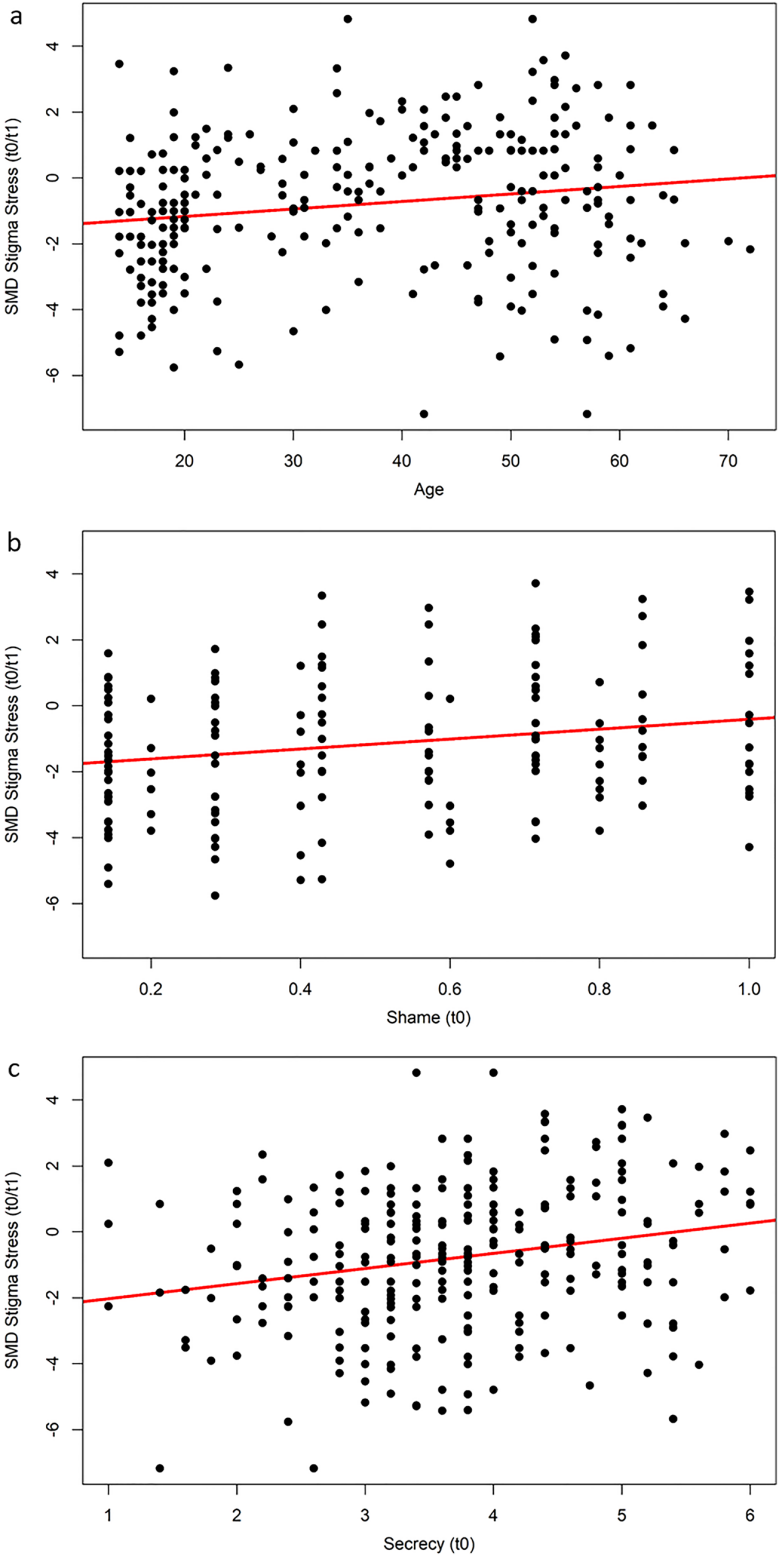
Three regressions on standardized mean differences (SMD) of stigma stress change from baseline (t0) to the end of HOP (t1) predicted by** a** age (in years),** b** shame at baseline, or** c** secrecy at baseline. SMD scores below zero indicate a reduction of stigma stress after HOP treatment (t1)

In exploratory single-predictor regressions, change in self-stigma was predicted by marital status (with lowest HOP effects on self-stigma among singles: b = 0.08, R^2^ = 0.02, p < 0.05) and HOP effects for self-stigma were stronger in the study with adolescents (b = − 0.07, R^2^ = 0.05, p < 0.05) and the study with suicide attempt survivors (b = − 0.18, R^2^ = 0.05, p < 0.01). Stronger pre-post reduction of self-stigma was predicted by greater empowerment at baseline (b = − 0.19, R^2^ = 0.03, p < 0.01; Fig. 1a); a parallel effect was found for shame, with more shame at baseline predicting less self-stigma reduction at the end of the HOP program (b = 0.18, R^2^ = 0.09, p < 0.01; Fig. 1b). In confirmatory regressions, pre-post stigma stress reduction was stronger for younger participants (b = 0.02, R^2^ = 0.03, p < 0.01; Fig. 2a), supporting our hypothesis for stigma stress as an outcome, but not for other outcomes. There was no effect of gender on any outcome (for gender see Supplementary Figs. 1 and 2).

Caucasian participants reported greater stigma stress decrease than Asians or other non-white participants (b = − 0.85, R^2^ = 0.03, p < 0.01) and likewise stigma stress reduction was weaker in the Hong Kong trial compared to all other trials (b = 1.28, R^2^ = 0.09, p < 0.001). Pre-post HOP effects on stigma stress were stronger for participants with lower education (b = − 1.32, R^2^ = 0.04, p < 0.01). Participants diagnosed with schizophrenia or psychosis reported less reduction in stigma stress pre-post compared to people with other diagnoses (b = 1.16, R^2^ = 0.07, p < 0.01). Higher levels of shame (b = 1.50, R^2^ = 0.04, p < 0.01; Fig. 2b) and secrecy (b = 0.46, R^2^ = 0.05, p < 0.001; Fig. 2c) at baseline predicted a lower pre-post reduction in stigma stress.

**Fig. 3:**
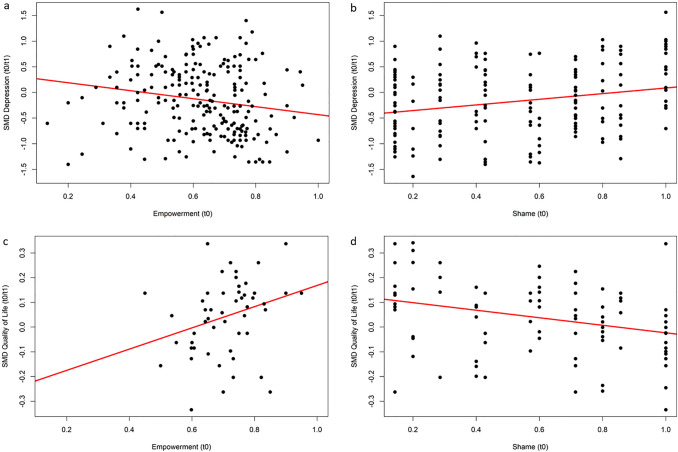
Regressions on standardized mean difference (SMD) of depressive symptom change (**a** and **b**) and quality of life (**c** and **d**) from baseline (t0) to end of HOP (t1), predicted by empowerment or shame at baseline (t0). Scores below zero indicate a reduction of depressive symptoms or quality of life, respectively, at t1

Improvement of depressive symptoms at t1 (pre-post) was stronger among participants with more empowerment (b = − 0.77, R^2^ = 0.03, p < 0.01; Fig. 3a) and less shame (b = 0.55, R^2^ = 0.05, p < 0.01; Fig. 3b) at baseline. A parallel pattern was found for HOP effects on quality of life (Figs. 3c and d): More empowerment (b = 0.43, R^2^ = 0.07, p < 0.05) and less shame (b = − 0.15, R^2^ = 0.08, p < 0.01) at baseline predicted greater improvements in quality of life at the end of the HOP program. For the importance of predictors across regression models, see Supplementary Fig. S1.

### Results of meta-regression analyses

For each of the four outcome domains (self-stigma, stigma stress, depressive symptoms, quality of life) a best meta-regression model was selected by *Glmulti*, with baseline variables as independent variables, and separately for change of outcome at t1 or at t2 as dependent variables, respectively (Table 1, upper and lower half).

**Table 1:** Results of best model selection via Glmulti for respective outcome changes between baseline (t0) and post-intervention (t1), upper half, or from baseline (t0) to follow-up (t2), lower half

Dependent variable (change from t0 to t1, or from t0 to t2)	Predictors in selected model	b	R^2^
Self-stigma (t1)	1. Shame (t0)	0.21***	0.19***
	2. Study: Mulfinger et al. [15] Sheehan et al. [20]	− 0.11*	
		− 0.18***	
	3. Marital status: Single	0.07*	
Stigma stress (t1)	1. Diagnosis of schizophrenia/psychosis (reference category: any other diagnosis)	1.16 **	0.07 **
Depressive symptoms (t1)	1. Shame (t0)	0.59**	0.08***
	2. Empowerment (t0)	− 0.74*	
Quality of life (t1)	1. Shame (t0)	− 0.15**	0.08**
Self-stigma (t2)	1. Shame (t0)	0.20***	0.11***
	2. Study: Mulfinger et al. [15] Rüsch et al. [19]	− 0.15***	
		− 0.08*	
Stigma stress (t2)	1. Educational level: University degree (reference category: high school degree)	1.25*	0.06*
	2. Shame (t0)	1.41*	
Depressive symptoms (t2)	1. Shame (t0)	0.57**	0.09***
	2. Empowerment (t0)	− 0.93**	
Quality of life (t2)	1. Secrecy (t0)	− 0.02*	0.03*

Predictors are arranged in order of importance to the specific regression model to predict standardized mean differences between baseline (t0) and end of HOP treatment (t1) or between baseline and follow-up (t2). *b* indicates the regression coefficient of each single predictor with scores above 0 indicating an increase in the respective SMD; a negative regression coefficient* b* for a single trial as predictor variable implies that participation in this trial predicted reduction in the respective outcome variable compared to participation in all other trials; R^2^ reflects variance explained by the regression model adjusted for number of predictors

*p < .05, **p < .01, ***p < .001

For change at t1 (post), the most important predictors across meta-regression models were shame (higher baseline shame predicting less benefit from HOP across three outcome domains: self-stigma, depressive symptoms, quality of life, see Table 1, upper half) and empowerment (higher baseline empowerment related to greater reduction of depressive symptoms). Study-level characteristics, socio-demographic variables and diagnosis also contributed to outcome variance in meta-regression models for change in self-stigma and stigma stress (Table 1, upper half).

Predictors of change at t2 (follow-up) were similar to predictors for change at t1 (Table 1, lower half): Again, higher shame at baseline was associated with less improvement (in self-stigma, stigma stress and depressive symptoms) at follow-up; a similar pattern applied to improvements in quality of life that was predicted by lower secrecy at baseline. Finally, participants with higher education reported less stigma stress reduction at follow-up. For the importance of predictors across regression models, see Supplementary Figure S2. Most of these findings stayed robust in sensitivity analyses, when imputed outcome scores at t2 were excluded from analyses (Supplementary Tables S3 and S4).

### Mediation analyses

**Fig. 4:**
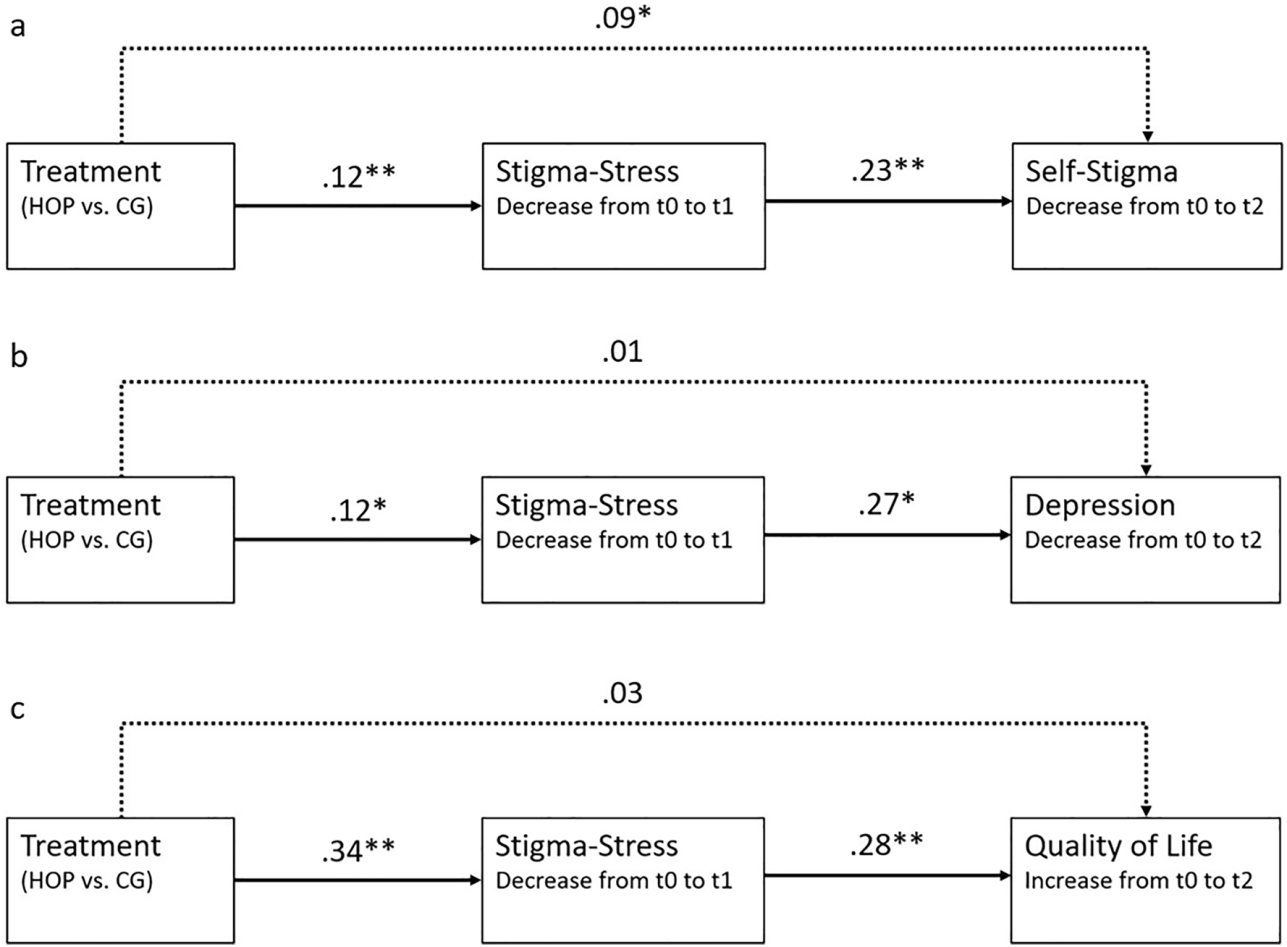
Mediation models for effects of HOP (relative to the control group/CG) on **a** self-stigma, **b** depression, or **c** quality of life at follow-up, mediated by reduced stigma stress at the end of the HOP program. Scores indicate regression coefficients b. *p < .05, **p < .01

Stigma stress reduction at t1 mediated positive HOP effects on self-stigma (b = − 0.03, p < 0.05; total effect: b = 0.12, p < 0.01; Fig. 4a) and on depressive symptoms (b = 0.03, p < 0.01; total effect: b = 0.05, p = 0.27; Fig. 4b) at follow-up (t2). Similarly, positive HOP effects on quality of life at t2 were mediated by reduced stigma stress at the end of HOP (t1; b = 0.10, p < 0.01; total effect: b = 0.12, p < 0.05; Fig. 4c). We found full mediation (with non-significant direct effects) for depressive symptoms and quality of life, and partial mediation (with a remaining weak, but significant direct effect) for self-stigma (Fig. 4).

### Risk of bias assessment

Results of the risk of bias assessment are provided in Supplementary Table S5. None of the included studies had a high risk of bias. Due to the nature of the HOP intervention, trials were unblinded for HOP participants, resulting in some concerns. Throughout all trials, the risk of bias in the randomization process and reporting bias were low.

## Discussion

Our meta-analysis, based on six RCTs with over 300 HOP participants, offers two main findings. First, those people with mental illness who were better off before the HOP program, in terms of more empowerment and less shame about their mental illness, had better HOP outcomes. Possibly, they found it easier not only to attend HOP groups in the first place, but also to engage in group discussions, to try out skills and strategies they learned in HOP, and to apply them in the real world. On the other hand, this finding highlights a possible challenge for the HOP intervention: Those most in need of support to cope with shame and self-stigma may not always benefit. A possible solution might be a more intense HOP version or an initial module to address illness-related shame before the start of the actual HOP program. This will be a fruitful area for future program development and evaluation, adapted to cultural contexts and different target groups and co-led by people with mental illness [33].

Second and confirming our hypothesis about reduced stigma stress at the end of the HOP program as mediator of HOP outcomes at follow-up, reduced stigma stress after HOP appears to be a key mechanism of change and a mediator of HOP effects on more distal outcomes over time. Those participants who, right after the HOP program, feel better prepared to cope with stigma and thus report less stigma stress, show benefits in a range of important outcome domains at follow-up: self-stigma, depressive symptoms and quality of life. The fact that reduced stigma stress leads to better quality of life during the follow-up period highlights the potential of HOP to improve outcomes beyond stigma variables, with high relevance for participants and society.

Despite nearly ten years of research, the evidence base for HOP is still limited. More data on different HOP versions and in particular about HOP’s effects over longer follow-up periods are needed. Given our findings in this meta-analysis it remains to be seen whether those participants with greater shame at baseline might catch up (or not) with others during the months following HOP. Newer HOP versions use a fourth booster session about a month after the third session; future research should examine its possible additional benefits compared to the original 3-session HOP version.

Our first hypothesis about younger age and female gender as predictors of better HOP outcomes was confirmed not for gender, but for age. Younger people likely still have to make more choices regarding their identity, social networks and disclosure and therefore might, on average, benefit more from the HOP program. A number of other individual-level variables that affected HOP outcomes appear relevant. First, it is good news that lower education predicted stronger stigma stress reduction: This means that HOP does not require a high education level to be effective. Second, the fact that people with a diagnosis of schizophrenia reported lower stigma stress reductions might be related to the fact that the public stigma of this disorder is stronger and has not decreased in the past decades, unlike the somewhat diminished public prejudice against people with common mental disorders [34]. This may make it more difficult for a program like to HOP to improve coping with public and self-stigma among people with schizophrenia. Finally, the potential role of ethnicity for HOP outcomes could not be fully explored in our study as three studies ([15, 19] and the unpublished Hong Kong trial) were conducted with ethnically homogeneous participants (all white in the Swiss and German studies, or all Chinese in Hong Kong, respectively).

The role of the cultural context could not be fully explored as only the trial in Hong Kong was conducted in a non-western country (which showed weaker HOP effects). Future studies should acknowledge the cultural milieu that may make HOP less effective in reducing stigma stress. In fact, through HOP, some participants might become more aware that stigma is highly prevalent in their society and in the environments in which they work and live, which may exacerbate their stress and reduce their disclosure efficacy and intentions. Instead of only looking at individual-level variables such as shame, the cultural/structural factors that may reinforce stigma and secrecy should be considered in future studies.

This study has a couple of other limitations. Despite the longitudinal RCT data, we cannot draw firm conclusions on causality. Deviating from the pre-registered PROSPERO protocol, we did not conduct a sensitivity analysis to compare the one-stage meta-analytical approach with a two-stage approach due to the low number of eligible studies. Younger participants might benefit more from HOP, but this remains speculative at this point with only one available HOP trial among adolescents [12, 15]. Finally, we could not examine whether the active ingredient of HOP is mainly its focus on disclosure decisions; or whether the fact that attending a peer support group (like HOP or others), which requires some degree of disclosure at least within the attended group, in itself may reduce self-stigma.

In summary, HOP as a brief peer-led group program may be especially helpful for those who are not extremely burdened by shame, lack of empowerment, or secrecy. Those who are, might benefit from a modified HOP version or a shame-reducing module beforehand. HOP’s positive effects on stigma stress as a proximal outcome likely lead to broader effects on self-stigma, symptoms and quality of life over time. This meta-analysis offers an empirical basis for the peer, advocacy and research communities in their joint work on the future development and evaluation of HOP as an important tool to help people with mental illness overcome self-stigma and shame.

### Supplementary Information

Below is the link to the electronic supplementary material.

Supplementary file1 (DOCX 227 KB)

## Data Availability

The original data of the six HOP trials were provided to the authors only for the scope of the current IPD analysis.
